# Percutaneous transhepatic biliary drainage as an effective complementary treatment option for clinically relevant biliopancreatic, pancreatic and biliary leakage after pancreaticoduodenectomy

**DOI:** 10.1186/s12893-026-04313-0

**Published:** 2026-11-04

**Authors:** Souheil Reda, Jon Unosson, Christopher Månsson, Selma Kabous, Angeliki Dimopoulou Creusen, Srinivas Sanjeevi, Petter Fruhling, Bengt Isaksson, Jozef Urdzik

**Affiliations:** 1 Department of Surgical Sciences, Gastrointestinal Surgery, Uppsala University, Uppsala, Sweden; 2 Department of Surgical Sciences, Radiology, Uppsala University, Uppsala, Sweden

**Keywords:** Pancreaticoduodenectomy, Percutaneous transhepatic biliary drainage, Postoperative pancreatic fistula, Biliopancreatic leakage

## Abstract

**Background:**

Clinically relevant postoperative pancreatic fistula (CR-POPF) and bile leakage (BL) are associated with high morbidity and mortality after pancreaticoduodenectomy (PD). Rescue pancreatectomy for CR-POPF after PD is also associated with major morbidity and mortality. Percutaneous transhepatic biliary drainage (PTBD) is a well-established treatment option for isolated bile leakage following PD. The efficacy of PTBD as a treatment option for clinically relevant biliopancreatic leakage after PD is still uncertain.

**Methods:**

Data from 543 patients scheduled for PD between 2014 and 2022 at Uppsala University Hospital, Sweden –– a tertiary referral centre –– were evaluated for clinically significant biliopancreatic, pancreatic or biliary leakage. Patients with unresolved leakage despite percutaneous intra-abdominal drainage were treated with PTBD. This study evaluates the technical success and clinical outcomes of this treatment.

**Results:**

PD was performed on 483 patients, of whom 83 (17%) had clinically relevant biliary, pancreatic or combined leakage. Thirty-nine (46%) of those with leakage received a PTBD, which was technically successful at the first procedure in 38 (97%) patients. Clinical success –– defined as discharge with closed PTBD, a resolved leak without the need for rescue pancreatectomy or in-hospital mortality –– was achieved in 32 (84%) patients. Two patients required an urgent rescue pancreatectomy without prior PTBD.

**Conclusion:**

PTBD appears to be an effective complementary treatment method for clinically relevant biliopancreatic leakage after PD. Although promising, PTBD-related complications are common, with 50% requiring drain revision, and thus the treatment should be considered with caution.

**​​Trial Registration:**

Not applicable. This​ ​is a retrospective registry-based observational study.

## Introduction

Pancreaticoduodenectomy (PD) is the cornerstone of and only curative treatment for malignant and premalignant tumours in the periampullary region and pancreatic head [1, 2]. Centralization to high-volume centres and optimization of perioperative care have decreased mortality rates to less than 3%, but associated morbidity remains high at 50% [1–3].

Postoperative pancreatic fistula (POPF) is a severe complication following PD resulting from a breakdown of the pancreatic anastomosis. POPF, as defined by the International Study Group of Pancreatic Fistula (ISGPF), is divided into clinically irrelevant biochemical leakage and clinically relevant POPF (CR-POPF) Grade B/C [4, 5]. The incidence of CR-POPF following PD is approximately 12%, with mortality rates up to 40% for Grade C fistulas [5–8].

The first line of treatment for biliopancreatic leakage after PD is generally conservative and followed, when necessary, by percutaneous catheter drainage near the dehiscent anastomosis. However, this may not always be sufficient. Further image-guided catheter drainage, somatostatin analogues and re-laparotomy are all commonly used and described in the literature. Re-laparotomy with rescue pancreatectomy is the most definitive treatment for POPF but is associated with up to 50% mortality [9]. One randomized trial advocated prophylactic pancreatectomy to lower the risk of postoperative morbidity and mortality, but it was criticized for its choice of morbidity as the primary endpoint and lack of patient-reported outcomes [10–12]. Theijse et al. noticed a significant increase in mortality and morbidity in patients developing CR-POPF compared to patients without CR-POPF, and the benefits of total pancreatectomy (TP) are rather small [13].

Percutaneous transhepatic biliary drainage (PTBD) is a well-established and recognized treatment option for bile leakage and biliary stricture following hepatobiliary and pancreatic surgery [14]. But PTBD as a treatment option for significant biliopancreatic leakage after PD has not been as widely reported. To the best of our knowledge, there is only one retrospective multi-centre study describing PTBD as a treatment option for biliopancreatic leakage after PD, and that study reported that it is effective in conjunction with percutaneous intra-abdominal drainage for clinically significant leakage [15].

The aim of the present observational study was to evaluate the safety, efficacy and timing of PTBD as a treatment option for clinically relevant leakage after PD.

## Methods

### Patients

All consecutive patients above 18 years of age who were scheduled for PD between January 2014 and December 2022 at Uppsala University Hospital were identified from the Swedish National Register for Pancreatic Cancer and patient medical records. The pancreatic registry is externally validated with prospectively collected data [16]. Patient medical records, cholangiogram and computed tomography (CT) images were also systematically reviewed in a pre-defined standardized form by a hepatopancreatobiliary surgeon. Because the national registry only began documenting postoperative pancreatic fistula (POPF) and bile leakage in May 2018, data from previous years had to be reviewed retrospectively. At that time, the alternative Fistula Risk Score (a-FRS) was not calculated systematically, as it is in current clinical practice at our department. For the present study, the a-FRS was retrospectively calculated for the entire study period for patients who received percutaneous transhepatic biliary drainage (PTBD), as well as for those who intraoperatively required conversion to total pancreatectomy due to technical factors encountered during surgery.

Patients undergoing PD during the study period received one passive drain placed dorsal to hepaticojejunostomy and pancreatojejunostomy (constructed using modified Blumgart technique) [17] and one passive drain ventral to pancreatojejunostomy and dorsal to gastrojejunostomy. Drain fluid output was analysed for amylase, whereas bilirubin measurement was not performed consistently particularly in the early study period; at that time (2014–2016), documented assessments were made by the treating surgeon. Conducting bilirubin measurement in drain fluid became a more consistent practice in the years after 2016. As a consequence of this, ISGLS definitions for bile leakage could not be applied retrospectively to the entire cohort [18]. POPF was defined as amylase activity at least three times higher than the institutional upper limit (3.3μkat/L) on postoperative day 3 or later. Likewise, a biliary leak was defined as a bilirubin concentration at least three times higher than the serum bilirubin concentration. Clinical relevance and POPF grade were defined according to ISGPS guidelines [5]. High- and low-volume fistulas were identified using a cut-off at 200 ml/day [4]. All patients with CR-POPF, mixed biliopancreatic leakage, or clinically relevant isolated bile leakage were treated with broad-spectrum antibiotics. Patients fulfilling the criteria for both POPF and bile leakage were classified as having a mixed biliopancreatic leak. CT findings and cholangiography performed during PTBD were also reviewed to determine the probable anatomical origin of the leakage. A mixed leak could therefore originate from separate pancreatojejunostomy and hepaticojejunostomy or both. If a mixed leak was noted, it was managed according to the same treatment strategy as POPF.

In cases of POPF or clinical suspicion of other postoperative complications, contrast-enhanced CT was performed. If a radiologically visible fluid collection was identified near the anastomosis, the position of the existing drain was adjusted, or additional percutaneous drainage was performed by an interventional radiologist to control the leakage. PTBD was considered in patients with persistent leakage and/or clinical deterioration despite optimization of intra-abdominal drainage.

**Fig. 1:**
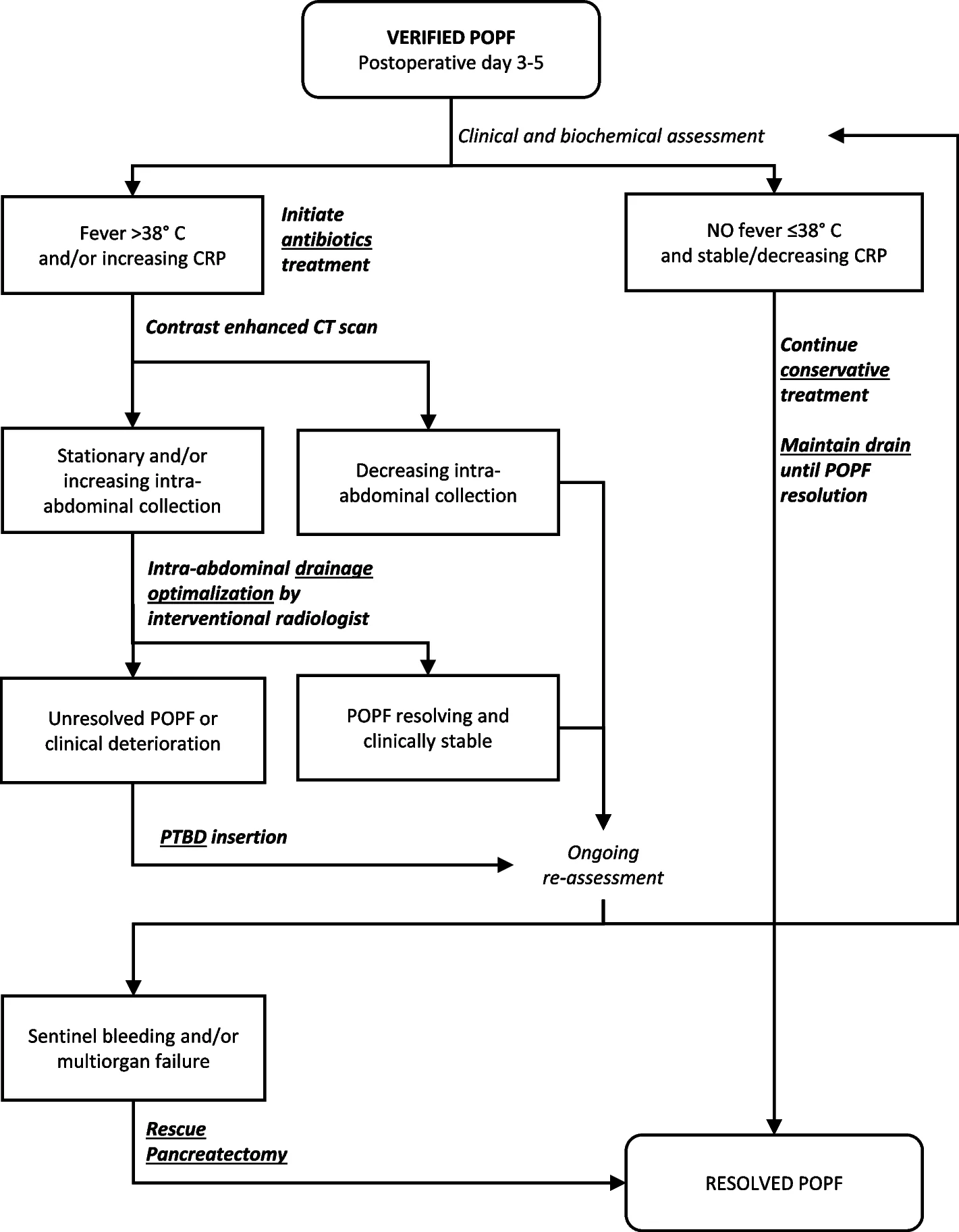
The step-up approach for POPF management. Systematic step-up approach was introduced in 2022, towards the end of the study period

During most of the study period (2014–2021), the decision to proceed with PTBD was based on the individual clinical assessment of treating surgeons rather than on a standardized treatment procedure. In 2022, a standardized stepwise approach was introduced (Fig. 1).

### Percutaneous transhepatic biliary drainage (PTBD) management protocol

**Fig. 2:**
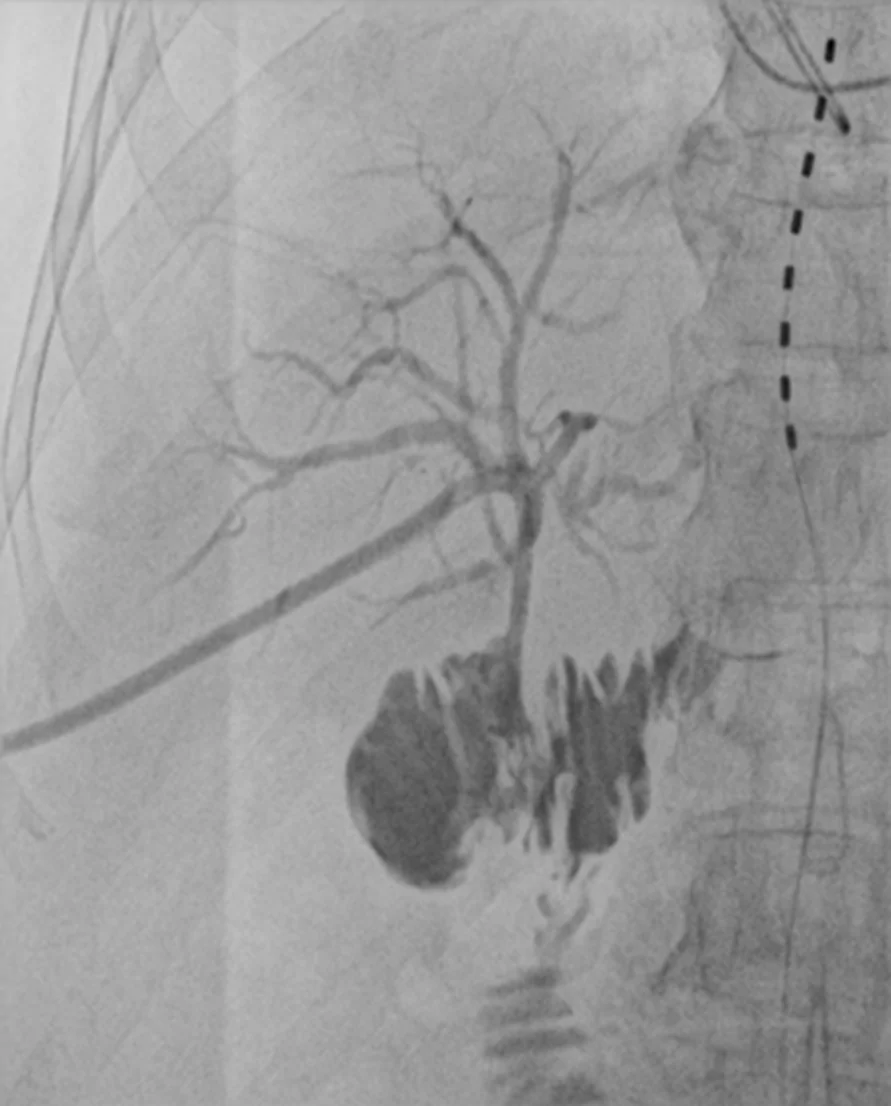
Percutaneous transhepatic biliary drainage (PTBD). PTBD was checked 10 days after insertion for mixed leak at the pancreaticojejunal anastomosis, with resolution of the POPF as observed. The control was made under fluoroscopy guidance

All procedures were performed in a Siemens angio suite, using either a Siemens ARTIS Pheno solution or a Siemens Artis Q (Siemens, Forchheim Germany). All patients were under general anaesthesia with the right arm extended from the body at a right angle. The lower part of the thorax and the upper part of the abdomen were disinfected, and the area demarcated with sterile drapes. The default choice of puncture was the right liver lobe, but the epigastric area was also sterile, should the need arise to puncture the left liver lobe. An ultrasound examination of the right flank was performed after the position of the right hemidiaphragm was identified by fluoroscopy. Usually, there are no ultrasound-visible bile ducts in the leakage setting, so the intrahepatic right portal vein was aimed at using a 20-gauge needle (Special cannula, Mediplast AB, Malmö, Sweden), with the tip directed either slightly cranial or caudal to the venous wall. Small amounts of contrast material were injected, and the needle retracted or forwarded until the bile duct was opacified. Efforts were made to inject as much contrast medium as needed to opacify the entire bile tree, if possible, the goal being to locate the anastomosis. A 0.018-inch Cope wire (Cook Medical, Inc., Bloomington, USA) was advanced aiming at the jejunal limb distal to the anastomosis. A Gould catheter introduction set was then used (Cook Medical, Inc., Bloomington, USA) and gradually advanced until the tip of the catheter was in the jejunal limb. The guide wire was then exchanged for a stiff 0.035-inch guide wire (Amplatz, Boston Scientific, Inc., Boston, USA), and either an 8.5 Fr or a 10.2 Fr multipurpose drainage catheter (Cook Medical, Inc., Bloomington, USA) was inserted, positioning the tip of the catheter towards the gastrojejunostomy. Side holes were manually cut in the body of the catheter corresponding to the biliary tree but not to the anastomosis. Transanastomotic placement into the jejunal limb was intended whenever technically feasible but could not be achieved in all procedures. The catheter was sutured to the skin and remained open, without any flush of saline, monitoring the output (Fig. 2).

### Outcome measures

Technical success was defined as the ability to place a PTBD during the first procedure even if several attempts had been made, and technical failure was defined as the need for a second procedure. Other technical outcomes were PTBD-related complications classified according to the Cardiovascular and Interventional Radiological Society of Europe (CIRSE) [19–21]. Major interventional complications were defined as Grade ≥ 3.

Clinical success was defined as hospital discharge with closed PTBD (drain left in situ but capped off) and resolution of the leak without further intervention related to the fistula. Clinical failure was defined as residual biliopancreatic, biliary or pancreatic leakage requiring further surgery, with rescue pancreatectomy being the standard salvage treatment, or patient death, post-PTBD within 90 days from index surgery, regardless of other complications. Demographic data, comorbidity, final diagnosis, need for any intervention after PTBD, need for rescue pancreatectomy, 30- and 90-day mortality were all assessed by reviewing patient medical records. To analyse the timing of PTBD insertion, the group was divided based on median post-operative day of insertion into an early and late PTBD insertion group.

The study was conducted with the approval of the Swedish Ethical Review Authority (Ref. 2024–03263-01, Uppsala County, Sweden) and in accordance with the Declaration of Helsinki.

### Statistical analysis

Descriptive univariate statistical methods were used for patient characteristics and outcomes**.** Data were presented as medians with interquartile ranges (IQRs) unless stated otherwise. The Mann–Whitney U-test or Kruskal–Wallis test was used for continuous variables and the Chi-squared test or Fisher´s exact test for categorical variables, depending on the expected cell counts. Statistical significance was set at *p*-value < 0.05 (two-sided). Statistical analyses were performed using IBM SPSS Statistics (version 29).

## Results

**Fig. 3:**
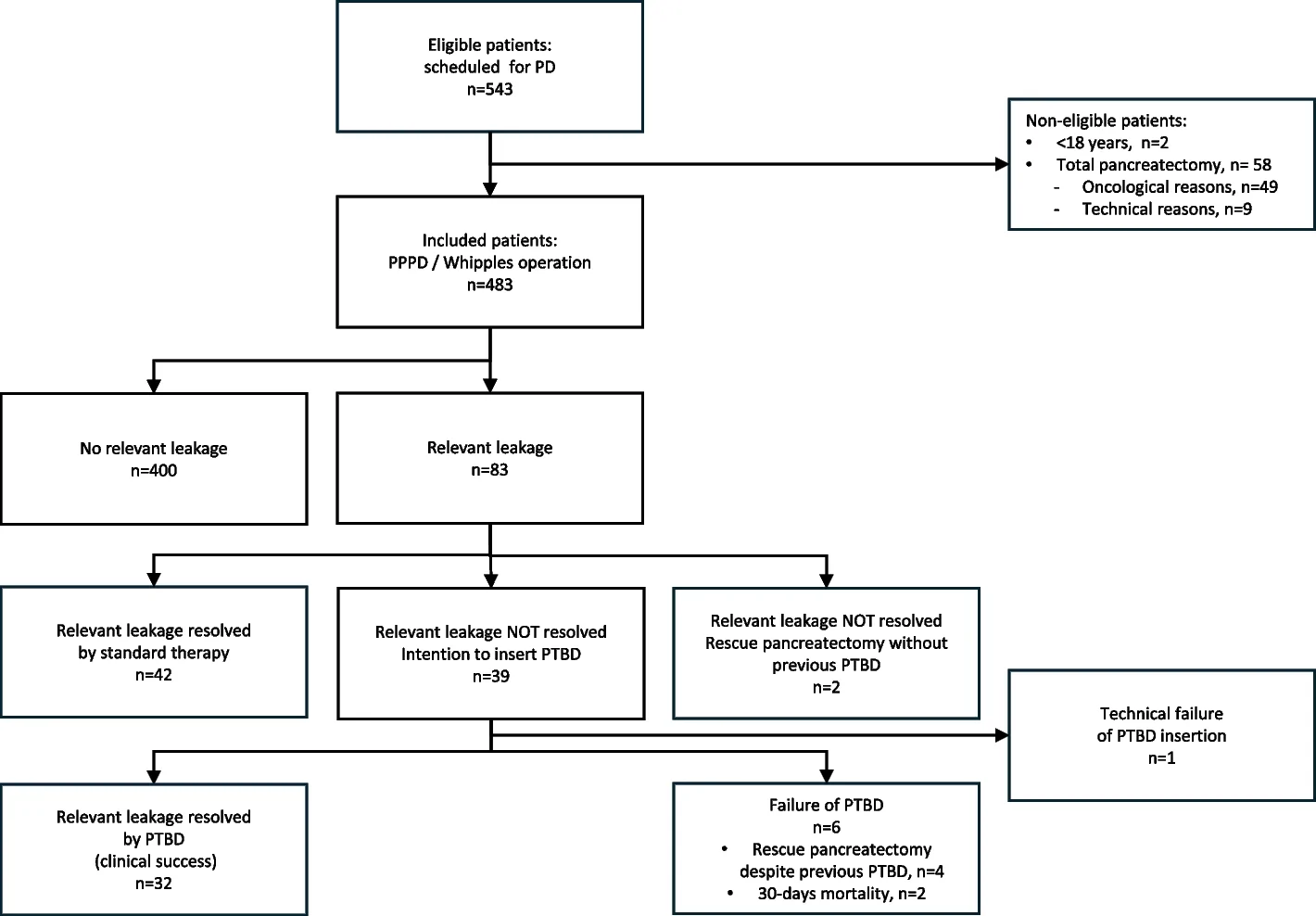
CONSORT diagram of patients included in the study

Between January 2014 and December 2022, 543 patients were scheduled for PD. Two patients younger than 18 years were excluded. For 58 patients (11%) the surgery was changed to a total pancreatectomy intra-operatively, 49 (9%) of them for oncological reasons and nine (1.7%) for technical reasons, such as a prophylactic pancreatectomy where the surgeon assessed the risk of POPF to be too high, with a median alternative Fistula Risk Score (a-FRS) of 25.3 [22]. (See CONSORT diagram in Fig. 3.)

### Patients undergoing pancreaticoduodenectomy

During the study period, 483 patients underwent PD. Patient characteristics are presented in Table 1. Four hundred patients (83%) had no clinically relevant leakage, including biochemical leak. Patients who developed clinically relevant leakage were less likely to have undergone preoperative biliary drainage (*p* < 0.001), had a higher BMI (*p* = 0.004), and were more likely to have benign histological findings (*p* = 0.006). However, at our institution, pancreaticoduodenectomy is performed only when malignancy or pre-malignancy is suspected preoperatively. Clinically relevant biliopancreatic leakage was also associated with higher 30-day mortality (p < 0.017), 90-day mortality (*p* = 0.001) and rescue pancreatectomy (*p* < 0.001).

**Table 1:** Characteristics of all patients who underwent PD

	All patients operated on with PD (*n* = 483)	No relevant leakage (*n* = 400)	Relevant leakage (*n* = 83)	*p*-value
Age (years) median [IQR]	70 [64–75]	70 [64–75]	69 [62–73]	0.188
Sex ratio (M:F)	242:241	199:201	43:40	0.810
BMI (kg/m^2^) median [IQR]	25 [23–28]	25 [23–28]	27 [24–29]	0.004
Obese	72 (15%)	54 (14%)	18 (22%)	0.063
ASA class				0.843
1	46 (10%)	36 (9%)	10 (12%)	
2	265 (55%)	220 (55%)	45 (54%)	
3	167 (35%)	140 (35%)	27 (33%)	
4	5 (0%)	4 (1%)	1 (1%)	
Type of histology *				0.006
Malignant	388 (80%)	331 (83%)	57 (69%)	
Premalignant or benign	94 (20%)	68 (17%)	26 (31%)	
Peri-operative bleeding (mL) median [IQR]	525 [350–1000]	525 [313–900]	525 [400–1000]	0.419
Pre-operative biliary drainage	284 (60%)	255 (64%)	29 (35%)	< 0.001
Pre-operative bilirubin (µmol/L) median [IQR]	12 [7–23]	13 [8–24]	9 [6–17]	0.006
Operation time (min) median [IQR]	417 [360–484]	417 [360–486]	415 [368–470]	0.895
Type of surgical procedure				0.393
Whipple	195 (40%)	158 (40%)	37 (45%)	
PPPD	288 (60%)	242 (60%)	46 (55%)	
Neoadjuvant treatment	18 (4%)	18 (5%)	0 (0%)	0.053
Rescue pancreatectomy	6 (1.2%)	0 (0%)	6 (7.2%)	< 0.001
30-day overall mortality	4 (0.8%)	1 (0.3%)	3 (3.6%)	0.017
90-day overall mortality	13 (2.7%)	5 (1.3%)	8 (9.6%)	< 0.001

Values are n (%) unless otherwise indicated. Obese was defined as BMI ≥ 30 kg/m^2^. M:F male:female. * Histology was unavailable for one patient. mL millilitres. Min minutes

### Patients with relevant leakage in the pancreaticojejunal or hepaticojejunal anastomosis

Among the 483 patients undergoing PD, 83 (17%) developed clinically relevant postoperative leakage. Sixty-nine (87%) were classified as ISGPS Grade B and 10 (13%) as Grade C with CR-POPF; the remaining four patients had isolated biliary leakage. Additional management consisted of broad-spectrum antibiotics and adjustment or additional placement of intra-abdominal drains. Resolution of leakage with initial management alone was more frequent among patients who had undergone preoperative biliary drainage (Table 2). In 39 patients with persistent leakage despite initial management, PTBD was subsequently attempted. Two patients underwent rescue pancreatectomy without prior PTBD. The first was performed on postoperative day 11 due to mixed biliopancreatic leakage with high volume fistula, where radiology revealed a dehiscence of the hepaticojejunostomy considered irreparable by PTBD insertion. The second was also due to a biliopancreatic leak, which developed into a life-threatening haemorrhage. The patient was taken to angiography with coiling of the branches of the superior mesenteric artery close to the pancreaticojejunal anastomosis but started to rebleed. Rescue pancreatectomy was performed on day 7 after index surgery. One of these two patients died within 30 days due to complications (Table 2).

**Table 2:** Characteristics of patients with relevant leakage

	Relevant leakage. Resolved with standard treatment (*n* = 42)	Relevant leakage Rescue pancreatectomy without PTBD (*n* = 2)	Relevant leakage Intention to insert PTBD (*n* = 39)	*p*-value	Relevant leakage resolved by PTBD (*n* = 32)	Relevant leakage NOT resolved by PTBD (*n* = 6)
Age (years) median [IQR]	69 [60–75]	74 [NA]	69 [65–73]	0.652	69 [65–73]	72 [67–75]
Sex ratio (M:F)	21:21	2:0	20:19	0.630	16:16	4:2
BMI (kg/m^2^) median [IQR]	26 [24–30]	27 [NA]	27 [24–30]	0.853	27 [24–30]	26 [23–32]
Obese	9 (21%)	0 (0%)	9 (23%)	1.000	7 (22%)	2 (33%)
ASA class				0.461		
1	5 (12%)	0 (0%)	5 (13%)		2 (6%)	2 (33%)
2	26 (62%)	2 (100%)	17 (44%)		16 (50%)	1 (17%)
3	10 (24%)	0 (0%)	17 (44%)		14 (44%)	3 (50%)
4	1 (2%)	0 (0%)	0 (0%)		0 (0%)	0 (0%)
Type of histology				0.408		
Malignant	28 (67%)	1 (50%)	28 (72%)		24 (75%)	3 (50%)
Premalignant or benign	14 (33%)	1 (50%)	11 (28%)		8 (25%)	3 (50%)
Peri-operative bleeding (mL) median [IQR]	575 [438–1003]	670 [NA]	500 [350–1000]	0.858	500 [350–1000]	800 [450–2150]
Preoperative biliary drainage	23 (56%)	1 (50%)	5 (13%)	< 0.001	3 (9%)	2 (33%)
Pre-operative bilirubin (µmol/L) median [IQR]	10 [7–18]	7 [NA]	9 [6–16]	0.424	9 [6–21]	10 [7–15]
Operation time (min) median [IQR]	427 [376–476]	427 [NA]	401 [359–464]	0.488	388 [359–460]	427 [358–678]
Type of surgical procedure				0.190		
Whipple	15 (36%)	1 (50%)	21 (54%)		18 (56%)	2 (33%)
PPPD	27 (64%)	1 (50%)	18 (46%)		14 (44%)	4 (67%)
Neoadjuvant treatment	0 (0%)	0 (0%)	0 (0%)		0 (0%)	0 (0%)
Rescue pancreatectomy	0 (0%)	2 (100%)	4 (10.3%)		0 (0%)	4 (67%)
30-day overall mortality	0 (0%)	1 (50.0%)	2 (5.1%)		0 (0%)	2 (33%)
90-day overall mortality	2 (4.8%)	1 (50.0%)	5 (12.8%)		2 (6%)	3 (50%)

Values are n (%) unless otherwise indicated. IQR is not reported for the rescue pancreatectomy group due to small sample size (*n* = 2). M:F male:female. Obesity was defined in BMI ≥ 30 kg/m^2^. mL millilitres. Min minutes

### Patients with relevant leakage and intended PTBD

Patients who received PTBD had a median a-FRS of 10 (IQR 4.2–16.1). No patient had dilated intrahepatic bile ducts at the time of PTBD insertion. The proportion of leakage types differed amongst those who received PTBD compared to those whose leakage resolved without PTBD (p < 0.001). Only four patients had isolated bile leakage and received a PTBD. In all these cases, the leak resolved without the need for rescue pancreatectomy or occurrence of in-hospital mortality. Thirty-five patients (90%) received PTBD because of CR-POPF. All four rescue pancreatectomy procedures after PTBD insertion were due to CR-POPF.

### Feasibility and technical outcomes of PTBD

Overall technical success was achieved in 38 out of 39 patients (97%), with the drainage reaching the jejunal limb in all cases. For the patient who did not receive PTBD at the first attempt, the fistula was ultimately resolved by modification of the passive drain without needing a second attempt at PTBD. In 34 of 38 patients, the biliary drain was placed through the right intrahepatic system, and in three patients through the left. In one case, a central puncture was performed, just cranial to the anastomosis. PTBD was inserted mostly in patients without previous biliary drainage (35 of 38 patients). Fourteen patients received an 8.5 Fr catheter and 24 patients a 10.2 Fr catheter. In 19 of 38 patients, one or more revisions of the PTBD were required (Table 3). The main reason for revision was the need to replace the existing drain with a higher calibre drain to improve drainage efficiency, due to dislocation or the drain becoming non-functional owing to intraluminal blockage. Of the 14 patients receiving 8.5F drain, 10 (71%) needed a revision.

**Table 3:** Feasibility and safety of PTBD for relevant leakage. Technical outcomes according to timing after PD

	Early PTBD < 12 POD (*n* = 18)	Late PTBD ≥ 12 POD (*n* = 20)	*p*-value
Puncture and route			0.192
Right intrahepatic duct	15	19	
Left intrahepatic duct	2	1	
Central bile duct	1	0	
Size of drain (French)			0.805
8.5 F	7	7	
10.2 F	11	13	
Previously not drained biliary system	16	19	0.486
Technical success after > 2 attempts in first procedure	5	3	0.931
Revision of PTBD	6	13	0.051
Major PTBD complication	3	8	0.113
Bleeding requiring intervention	2	4	
Sepsis	0	2	
Multi-organ failure	1	2	
Minor PTBD complication	5	4	0.573
Intrahepatic haematoma	2	0	
Cholangitis	3	4	

Values are n unless otherwise indicated. F, French

### Safety of PTBD

Major complications after PTBD, defined as Grade ≥ 3 according to CIRSE guidelines, occurred in 11 of 38 patients, most commonly due to bleeding, which required intervention in six cases (Table 3). Four of six patients requiring intervention developed haemobilia requiring embolization of an arterial branch. The other two interventions were due to bleeding pseudoaneurysm from the proper hepatic artery and bleeding from the gastroduodenal artery stump. These events occurred after PTBD insertion, however a direct causal relationship with PTBD could not be established with certainty. Minor complications occurred in nine patients, most commonly cholangitis without organ failure or intensive care admission. Two patients died after PTBD insertion but before rescue pancreatectomy was performed. The first patient died due to major post-procedure haemorrhage after PTBD insertion, which was directly related to the procedure. The second patient had an early PTBD insertion, which controlled the CR-POPF. PTBD closure was possible on postoperative day 7, but the patient developed massive superior mesenteric vein thrombosis and subsequent intestinal ischemia and died on postoperative day 23.

### Efficacy of PTBD

The overall clinical success rate of PTBD insertion was 32 of 38 cases (84%) (Fig. 3 and Table 2). Demographic characteristics were similar amongst those whose leakage was resolved by PTBD compared to the group with PTBD failure (Table 2). Among the latter group, four patients had a rescue pancreatectomy and were later discharged from hospital without leakage. The median number of postoperative days for rescue pancreatectomy after primary surgery was 27. Three out of four cases had a low-volume fistula. The four patients undergoing rescue pancreatectomy after PTBD received PTBD more than 12 days postoperatively.

## Timing of PTBD and outcome

Timing of PTBD insertion depended on the patient’s clinical status and fistula control, with a median time to PTBD of 12 days post-surgery (IQR 7–16). For descriptive analysis, patients were assigned to the early (< 12 days) or the late (≥ 12 days) PTBD insertion group. Demographic characteristics and type of leakage were equally distributed between groups (Table 4). Early PTBD insertion occurred primarily for uncontrolled high-volume fistulas (13 of 18 cases). Late PTBD insertion occurred primarily for persistent low-volume fistulas (15 of 20 cases). The timing of PTBD seems to have been dependent on the volume output of the fistula (*p* = 0.004) rather than on a clinical step-up procedure.

**Table 4:** Efficacy of PTBD for relevant leakage. Indications and results according to timing after PD

	Early PTBD < 12 POD (*n* = 18)	Late PTBD ≥ 12 POD (*n* = 20)	*p*-value
a-FRS median [IQR]	7.0 [3.8–13.0]	11.2 [4.7–20.2]	0.519
Perioperative bleeding (ml) median [IQR]	500 [400–1000]	600 [320–1000]	0.509
Type of relevant leakage			0.890
Isolated CR-POPF	6	7	
Mixed biliopancreatic leak	10	11	
Isolated bile leak	2	2	
Fistula volume			0.004
High	13	5	
Low	5	15	
PTBD closure on (days) median [IQR]	15 [12–24]	34 [25–52]	0.252
Rescue pancreatectomy	0	4	0.045
ICU stay (days) median [IQR]	0 [0–2]	0 [0–9]	0.330
30-day mortality	2	0	0.126
90-day mortality	2	3	0.270

Values are n unless otherwise indicated. a-FRS = Alternative Fistula Risk Score. ICU, intensive care unit. 39 patients underwent attempted PTBD. 38 patients had successful PTBD placement and are included in Table 4

## Discussion

CR-POPF remains a major complication after PD and is associated with significant morbidity and mortality. PTBD is a feasible adjunct treatment option and a complement to the conservative approach and passive drainage. In isolated POPF, PTBD does not directly drain the pancreatic fistula, rather the biliary diversion reduces biliary flow through the jejunal limb, which may facilitate fistula healing. In mixed biliopancreatic leakage, PTBD additionally provides direct biliary diversion, when the biliary component is present. Although all patients presented with a non-dilated biliary system, a 97% technical success rate was achieved but with the need for revision in 50% of cases, which is in line with other reports [15, 23], emphasizing the need for an experienced multidisciplinary team to ensure optimal management. A higher revision rate was observed with 8.5Fr catheters (10/14) compared with 10.2 Fr catheters. This emphasizes the technical challenge associated with a non-dilated bile duct and the subsequent need for replacement with a larger calibre catheter.

Known risk factors such as higher BMI or benign pathology, verified in the cohort, are associated with softer pancreatic texture and a higher risk of anastomosis leakage [24]. In our cohort, preoperative biliary drainage was associated with a lower incidence of clinically relevant leakage and higher resolution rate without PTBD (Table 1). This is contrary to results from the randomized trial by Van der Gaag et al. [25], in which routine pre-operative biliary drainage had a higher incidence of severe post-operative complications (Clavien-Dindo ≥ 3 A). Therefore, the observed association should not be interpreted as evidence of a protective effect of preoperative biliary drainage. The discrepancy may reflect differences in patient selection as well as residual confounding due to the retrospective design of our study. Smits et al. reported a reduction in 90-day mortality and major postoperative complications when applying a step-up procedure for early recognition and management of complications [26]. This supports the potential value of a structured treatment procedure. In our cohort, fewer major complications were observed among patients undergoing early PTBD (Table 3). However, early and late PTBD were defined by median time rather than a predefined clinical threshold and were based on individual surgeon assessments rather than on our step-up procedure. For this reason, no conclusion could be drawn, although the observed association is interesting and consistent with the findings of Smits et al. [26].

The absence of difference in 90-day mortality in our cohort in relation to clinically relevant postoperative leakage irrespective of PTBD may be due to the small number of events. Overall, 90-day mortality in the entire cohort was 2.7%, while 30-day mortality among patients with clinically relevant leakage was 3.6%. The range we observed is similar to the in-hospital mortality reported in a recent study on a Dutch cohort [13]. Early PTBD insertion was significantly higher in patients with a high-volume fistula (Table 4). This suggests that, at our department, fistula volume influences the threshold to PTBD insertion. Patients with a high-volume fistula were more likely to undergo earlier escalation to PTBD. This is contrary to low-volume fistulas and late PTBD insertion, where we adjust the intra-abdominal drainage and take a careful wait-and-see approach. One of the patients mentioned in the results section died as a direct consequence of PTBD. Amongst the 483 patients operated on during the study period, rescue pancreatectomies were performed on only six (1.2%). Of these, four patients received PTBD, which was kept after the surgery, and all were subsequently discharged from hospital; three were alive at 90 days. Previous studies have reported clinical success rates of 70–96% following PTBD [15, 19, 23, 27, 28]. Of these studies, only Henry et al. specifically reported results for PTBD after pancreaticoduodenal surgery, showing a clinical success rate of 94% [15]. Mosconi et al. noticed that leakage after pancreaticoduodenectomy was associated with a greater need for re-intervention and higher occurrence of death than was leakage after liver resections when treated with PTBD insertion for bile leakage [23]. Optimization of intra-abdominal drainage and a conservative approach are still considered the gold standard option for CR-POPF [8, 15, 23], and for all patients receiving a PTBD there was an attempt to optimize the passive drainage before or during PTBD insertion.

The present single-centre retrospective cohort study has several limitations. First, the PTBD cohort represents a highly selected group of patients with persistent postoperative leakage despite initial conservative management and optimization of intra-abdominal drainage. The absence of a control group precludes conclusions regarding the superiority of PTBD over other treatment strategies, and the observed clinical success rate should be interpreted as a descriptive outcome of our cohort. Furthermore, during the long study period, management practices changed. During 2014–2021, which comprised most of the cohort, the decision to proceed with PTBD was based on individual clinical assessment rather than a standardized treatment procedure, the latter approach having been introduced in 2022. This clinical variation and retrospective selection of patients for PTBD may have introduced selection bias. In the only patient for whom PTBD insertion failed technically, the leakage resolved following optimization of intra-abdominal drain prior to a second PTBD attempt. This shows that further optimization of intra-abdominal drainage may be sufficient in selected patients and further limits the conclusion. Another limitation concerns the classification of biliary leakage. During the early study period (2014—2016), drained bilirubin was not routinely measured at our department, only drained amylase, which precludes providing a consistent ISGLS definition for the entire cohort. This may have led to a false low mixed leak proportion but did not appear to have influenced the incidence of CR-POPF. Detailed classification of leakage type was primarily performed in patients considered for PTBD.

The proportion of clinically relevant leakage in the whole cohort is 17%. This must be viewed in light of the fact that the addition of antibiotics created a larger group with type B leakage compared to previous definitions of POPF grading, as shown by Pulvirenti et al. [29].

## Conclusion

In summary, the present study is one of few reports evaluating PTBD as a complement to intra-abdominal drainage for clinically relevant postoperative leakage after PD. PTBD is a feasible complementary treatment option for persistent biliopancreatic leakage after PD when a conservative approach and intra-abdominal drainage are not sufficient. However, PTBD is a technically demanding procedure associated with substantial risk of major complications (29%) and frequent need for catheter revision (50%). Given the retrospective design, absence of a control group and patient selection, these findings should be interpreted with caution and require validation in larger prospective studies.

## Data Availability

The datasets generated and/or analyzed during the current study are available from the corresponding author on reasonable request.

## Abbreviations

a-FRS: Alternative fistula risk score
ASA: American Society of Anesthesiologists
BMI: Body mass index
CR-POPF: Clinically relevant postoperative pancreatic fistula
IQR: Interquartile range
n: Count number
PD: Pancreaticoduodenectomy
PPPD: Pylorus preserving pancreatoduodenectomy
PTBD: Percutaneous transhepatic biliary drainage
SD: Standard deviation
Whipple: Pylorus resecting pancreatoduodenectomy
